# Is Increased Susceptibility to Balkan Endemic Nephropathy in Carriers of Common *GSTA1* (*A/*B) Polymorphism Linked with the Catalytic Role of GSTA1 in Ochratoxin A Biotransformation? Serbian Case Control Study and *In Silico* Analysis

**DOI:** 10.3390/toxins6082348

**Published:** 2014-08-08

**Authors:** Zorica Reljic, Mario Zlatovic, Ana Savic-Radojevic, Tatjana Pekmezovic, Ljubica Djukanovic, Marija Matic, Marija Pljesa-Ercegovac, Jasmina Mimic-Oka, Dejan Opsenica, Tatjana Simic

**Affiliations:** 1 Institute of Medical and Clinical Biochemistry, Faculty of Medicine, University of Belgrade, 11000 Belgrade, Serbia; E-Mails: panlabkv@yahoo.com (Z.R.); asradojevic@tehnicom.net (A.S.-R.); marija_opacic@yahoo.com (M.M.); marijaercegovac@med.bg.ac.rs (M.P.-E.); okasn@rcub.bg.ac.rs (J.M.-O.); 2 Faculty of Chemistry, University of Belgrade, 11000 Belgrade, Serbia; E-Mail: mario@chem.bg.ac.rs; 3 Faculty of Medicine, University of Belgrade, 11000 Belgrade, Serbia; E-Mail: pekmezovic@sezampro.rs; 4 Institute of Epidemiology, Faculty of Medicine, University of Belgrade, 11000 Belgrade, Serbia; 5 Clinic of Nephrology, Clinical Center of Serbia, 11000 Belgrade, Serbia; E-Mail: ljubicadjukanovic@yahoo.com; 6 Institute of Chemistry, Technology, and Metallurgy, University of Belgrade, 11000 Belgrade, Serbia

**Keywords:** Balkan endemic nephropathy, biotransformation, glutathione transferase A1, ochratoxin A, polymorphism

## Abstract

Although recent data suggest aristolochic acid as a putative cause of Balkan endemic nephropathy (BEN), evidence also exists in favor of ochratoxin A (OTA) exposure as risk factor for the disease. The potential role of xenobiotic metabolizing enzymes, such as the glutathione transferases (GSTs), in OTA biotransformation is based on OTA glutathione adducts (OTHQ-SG and OTB-SG) in blood and urine of BEN patients. We aimed to analyze the association between common *GSTA1*, *GSTM1*, *GSTT1*, and *GSTP1* polymorphisms and BEN susceptibility, and thereafter performed an *in silico* simulation of particular GST enzymes potentially involved in OTA transformations. *GSTA1*, *GSTM1*, *GSTT1* and *GSTP1* genotypes were determined in 207 BEN patients and 138 non-BEN healthy individuals from endemic regions by polymerase chain reaction (PCR). Molecular modeling *in silico* was performed for GSTA1 protein. Among the GST polymorphisms tested, only *GSTA1* was significantly associated with a higher risk of BEN. Namely, carriers of the GSTA1*B gene variant, associated with lower transcriptional activation, were at a 1.6-fold higher BEN risk than those carrying the homozygous GSTA1*A/*A genotype (OR = 1.6; *p* = 0.037). In *in silico* modeling, we found four structures, two OTB-SG and two OTHQ-SG, bound in a GSTA1 monomer. We found that *GSTA1* polymorphism was associated with increased risk of BEN, and suggested, according to the *in silico* simulation, that GSTA1-1 might be involved in catalyzing the formation of OTHQ-SG and OTB-SG conjugates.

## 1. Introduction

**Chart 1:**
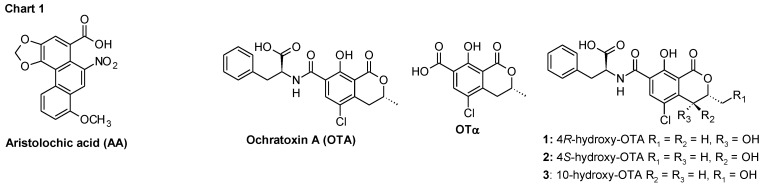
Structures of aristolochic acid (AA), ochratoxin (OTA) and its metabolites.

**Chart 2:**
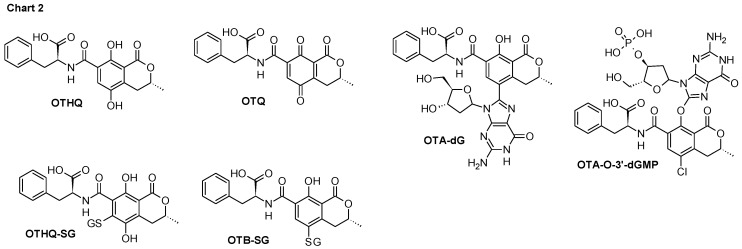
Ochratoxin (OTA) metabolites and OTA-specific DNA adducts (OTA-dG and OTA-O-3'-dGMP); OTHQ-ochratoxin hydroquinone, OTQ-ochratoxin quinone, OTHQ-SG-ochratoxin hydroquinone conjugated with glutathione.

Balkan endemic nephropathy (BEN) is a chronic tubulointerstitial kidney disease. This nephropathy is recognized as endemic in certain rural areas of Serbia, Bosnia, Croatia, Bulgaria, and Romania [1]. There is an ongoing discussion in the literature whether aristolochic acid (AA) or ochratoxin A (OTA) (Chart 1) are linked to BEN. In favor of the hypothesis that environmental exposure to OTA is a significant risk factor for the development of the disease is the detection of OTA-specific DNA adducts OTA-dG and OTA-O-3'-dGMP (Chart 2) in BEN patients from Serbia, Croatia, and Bulgaria [2,3], as well as in Bulgarian farmer suffering from BEN and upper urinary tract tumors [3]. One of the common features of BEN is that not all individuals exposed to OTA suffer from nephropathy and cancer [4], suggesting individual susceptibility as a risk factor. Besides differences in the cumulated dose of OTA and the duration of OTA intake, differences in OTA detoxification could be the reason for this individual susceptibility. Many genes of enzymes metabolizing xenobiotics are known to exist in variant forms of polymorphisms and appear to be important determinants of risk of diseases [5]. Thus, the identification of enzymes principally involved in the metabolism (*i.e.*, activation and/or detoxification) of OTA in humans and detailed knowledge of their catalytic specificities is of major importance.

The metabolic fate of OTA in both animals and humans evolved in two directions. One, which comprises hydrolysis [6] (ochratoxin α-OTα, Chart 1) and oxidations [7] (Products 1, 2, 3, Chart 1). The second pathways comprises bioactivation, which could lead either to detoxification or to genotoxicity by this mycotoxin [8]. There is strong evidence suggesting a key role of carboxypeptidase A, which converts OTA into its non-toxic metabolite OTα and phenylalanine. However, it seems that due to the slow metabolic rate, a small portion of OTA undergoes transformation into hydroquinone and quinone derivatives (OTHQ and OTQ respectively, Chart 2) after bioactivation. Bioactivation of OTA into OTHQ/ OTQ derivatives is supposed to be mediated by CYP1A1 and 3A4 in the liver [8], and probably by CYP3A5 in the kidneys [9]. They are further transformed into a glutathione conjugate (OTHQ-SG, Chart 2), as a result covalent bond formation between C6 of OTQ and a thiol-group of the reduced glutathione. Existence of this pathway in humans was supported by evidence of OTHQ-SG presence in blood and urine samples of BEN patients. Furthermore, in addition to OTHQ-SG, very recently the presence of OTB-SG (Chart 2) conjugates has also been shown in this biological material [7,10].

While both OTHQ-SG and OTB-SG appear to be stable markers of OTA exposure, it remains controversial whether these GSH metabolites are more or less toxic than OTA itself. It should be emphasized that both reactions, which result in the formation of OTHQ-SG and OTB-SG conjugates could be catalyzed by members of the glutathione transferases (GST) family. Despite the fact that GSTs might be involved in various pathways of OTA metabolism and make up 3%–5% and 1% of the total protein in the liver and kidney, respectively [11], the distinct role of these enzymes in OTA metabolism, especially with regard to BEN susceptibility, has been underexplored.

The family of cytosolic GSTs is comprised of different classes, including Alpha (GSTA), Mu (GSTM), Pi (GSTP), and Theta (GSTT) classes. The active site can be divided into two sub-sites: a polar site for binding GSH (G-site) and an adjacent hydrophobic region for electrophile substrates (H-site) [12]. GSTs show different abilities to bind xenobiotics, from promiscuity to high specificity. Approximately half of the general population lacks GSTM1-1 enzyme activity, due to a homozygous deletion of the *GSTM1* gene [13]. In the case of the *GSTT1* gene, homozygous deletion is present in about 20% of Caucasians, resulting in the lack of GSTT1-1 enzyme activity [14]. Single-nucleotide polymorphism (SNP), leading to amino acid substitution from isoleucine (Ile) to valine (Val) [15], changes the catalytic activity of the GSTP1-1 enzyme [16]. *GSTA1* polymorphism is represented by three, apparently linked, SNPs, which result in differential expression with lower transcriptional activation of the variant GSTA1*B (-567G, -69T, -52A) than the common GSTA1*A allele (-567T, -69C, -52G) [17]. Since GSTs have potential to conjugate OTA and its metabolites on one hand, and reduce free radicals that originate from OTA metabolites on the other, it is possible that *GST* genotype variations, with consequential absent or lower enzyme activities, may modify susceptibility to BEN. So far, the data on association between BEN and *GST* polymorphisms are scarce. This has prompted us to assess whether the common polymorphisms in *GSTA1*, *GSTM1*, *GSTT1*, and *GSTP1* are associated with susceptibility to BEN.

We found that *GSTA1* polymorphism was associated with increased risk of BEN, and examined *in silico* the capabilities of GSTA1 for acceptance of large substrates like OTHQ and reactive OTA metabolite into H-site and production of OTHQ-SG and OTB-SG conjugates. As result it was suggested that GSTA1-1, as the most promiscuous GST enzyme might be involved in the formation of OTHQ-SG and OTB-SG conjugates.

## 2. Results and Discussion

Demographic characteristics of patients and controls included in the study are shown in Table 1. As presented, there were no statistically significant differences with respect to age and gender.

**Table 1:** Demographic characteristics of Balkan endemic nephropathy (BEN) cases and controls.

Demographic characteristics	Cases	Controls	*p* value
Sex			0.134
Male *n*(%)	116 (56)	66 (48)	
Female *n*(%)	91 (44)	72 (52)	
Age			
Mean ± SD	70.60 ± 6.54	69.33 ± 9.98	0.153

*n*, number of cases or controls.

The distribution of frequencies of *GST* genotypes in BEN patients and controls is presented in Table 2. The obtained distribution of allelic frequencies of *GSTA1* and *GSTP1* genes was consistent with the Hardy-Weinberg equilibrium (HWE) in both cases and controls (*GSTA1*: *p* = 0.429 for cases, *p* = 0.135 for controls; *GSTP1*: *p* = 0.539 for cases, *p* = 0.521 for controls). On the other hand, in the case of *GSTM1* and *GSTT1* polymorphisms, HWE was not determined, since the methods used could not distinguish between homozygous and heterozygous genotypes. The results obtained showed that *GST* genotype distribution among healthy subjects in endemic regions did not differ from those reported in healthy subjects living in non-endemic regions [18,19]. When the frequency of *GSTA1*, *GSTM1*, *GSTP1*, and *GSTT1* genotypes was analyzed in healthy subjects who lived in endemic regions and compared to those in healthy subjects who lived in non-endemic regions, no significant difference was obtained (data not shown).

**Table 2:** *GSTA1*, *GSTM1*, *GSTT1* and *GSTP1* genotypes in relation to the risk of BEN.

Genotypes of GST	Cases *n* (%)	Controls *n* (%)	OR ^a^ with 95% CI	*p* value
*GSTA1 C-69T*				
*A/*A	58 (28)	53 (39)	1.0 ^b^	
*A/*B	110 (53)	57 (41)	1.8 (1.1–2.9)	0.021
*B/*B	39 (19)	28 (20)	1.3 (0.7–2.4)	0.377
*A/*B *+* *B/*B	149 (72)	85 (61)	1.6 (1.0–2.6)	0.037
*GSTM1*				
*active* ^c^	115 (56)	75 (54)	1.0 ^b^	
*null* ^d^	92 (44)	63 (46)	0.9 (0.6–1.5)	0.790
*GSTT1*				
*active* ^c^	168 (81)	100 (72)	1.5 (0.9–2.5)	0.126
*null* ^d^	39 (19)	38 (28)	1.0 ^b^	
*GSTP1* *A1578G*				
*Ile*/*Ile*	97 (47)	62 (45)	1.0 ^b^	
*Ile*/*Val*	92 (45)	57 (42)	1.0 (0.6–1.6)	0.953
*Val*/*Val*	16 (8)	18 (13)	0.6 (0.3–1.2)	0.142
*Ile*/*Val* + *Val*/*Val*	108 (53)	75 (55)	0.9 (0.6–1.4)	0.666

*n*, number of cases or controls; OR, odds ratio, 95%; CI, confidence interval; ^a^ adjusted by age and gender; ^b^ reference group; ^c^ *active* if at least one active allele present; ^d^ *null* if no active allele present.

As presented in Table 2, a significant association between the *GST* genotype and the risk of BEN development was found only for the *GSTA1* polymorphism. Namely, individuals with *GSTA1* *A/*B or *B/*B genotype were at a 1.6-fold higher risk of BEN than individuals carrying the homozygous GSTA1*A/*A genotype (OR = 1.6; CI = 1.0–2.6; *p* = 0.037).

Regarding *GSTM1* polymorphism, no significant difference between *active/null* genotype distribution among cases and controls was obtained. Thus, individuals carrying *GSTM1* *null* genotype were at a similar risk of BEN as those with *GSTM1* *active* genotype (OR = 0.9; CI = 0.6–1.5; *p* = 0.790).

Although in the case of *GSTT1* polymorphism, the frequency of the *GSTT1* *active* genotype was higher in BEN patients than in controls (81% *vs.* 72%, respectively, *p* = 0.057) and the risk of BEN was 1.5 times higher in individuals with *GSTT1* *active* genotype in comparison to *GSTT1* *null* individuals, statistical significance in terms of BEN risk was not reached (OR = 1.5; CI = 0.9–2.5; *p* = 0.126).

Results of *GSTP1* polymorphism in patients with BEN have shown lack of association between *GSTP1* genotype and risk of BEN. The wild-type *GSTP1* genotype (*Ile*/*Ile*) was found as the most common in both patients and controls. Although in BEN patients, the frequency of the *Val*/*Val* genotype was slightly lower in comparison to controls (8% *vs.* 13%, respectively) and it seemed that this allele might have some effect on decreasing the risk of BEN, statistical significance was not reached (OR = 0.6; CI = 0.3–1.2; *p* = 0.142).

We further analyzed the combined effect of *GST* genotypes on the risk of BEN. The results obtained show that when *GSTA1* genotypes are analyzed in combination with *GSTT1* genotypes (Table 3), the highest risk of BEN occurrence is obtained in homozygous and heterozygous carriers of mutant GSTA1*B allele with the *GSTT1* *active* genotype (OR = 2.3; CI = 1.0–5.3; *p* = 0.046).

**Table 3:** Combined effects of *GSTA1* with *GSTT1* genotypes in relation to the risk of BEN.

Genotypes	*GSTA1*	
	*A/*A	*A/*B+*B/*B
*GSTT1 active* ^a^		
Ca/Co	46/38	122/62
OR ^b^ (95% CI)	1.4 (0.6–3.4)	2.3 (1.0–5.3) ^c^
*GSTT1 null* ^d^		
Ca/Co	12/15	27/23
OR ^b^ (95% CI)	1.0 ^e^	1.5 (0.6–3.9)

OR, odds ratio, 95%; CI, confidence interval; Ca, number of patients; Co, number of controls; ^a^ *active* if at least one active allele present; ^b^ Adjusted by age and gender; ^c^ Statistically significant difference when compared with the reference group; ^d^ *null* if no active allele present; ^e^ reference group.

**Figure 1:**
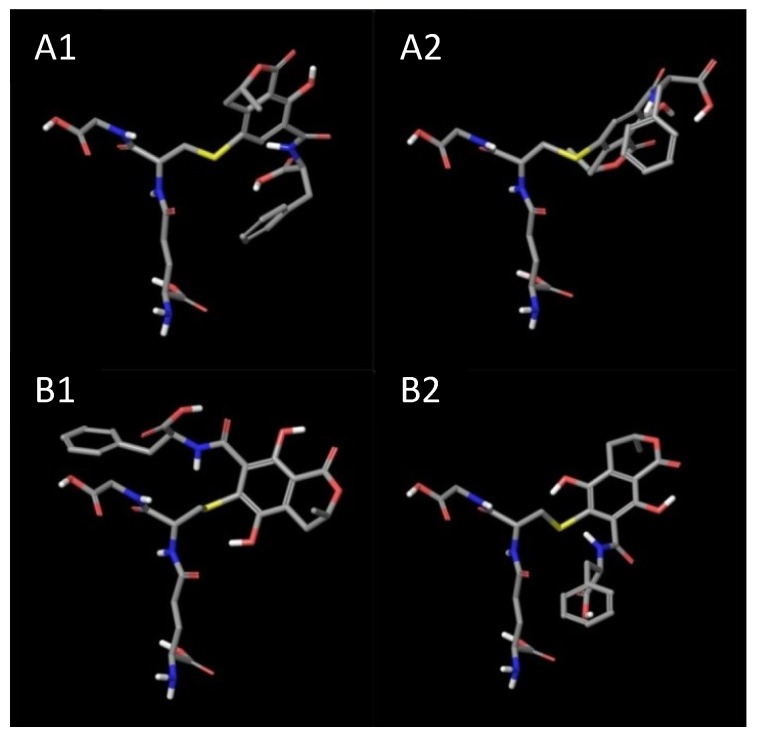
Three dimensional (3D) structures of the glutathione bound OTA in glutathione transferase A1-1. Possible structures of OTB-SG (A1 and A2), possible structures of OTHQ-SG (B1 and B2).

**Figure 2:**
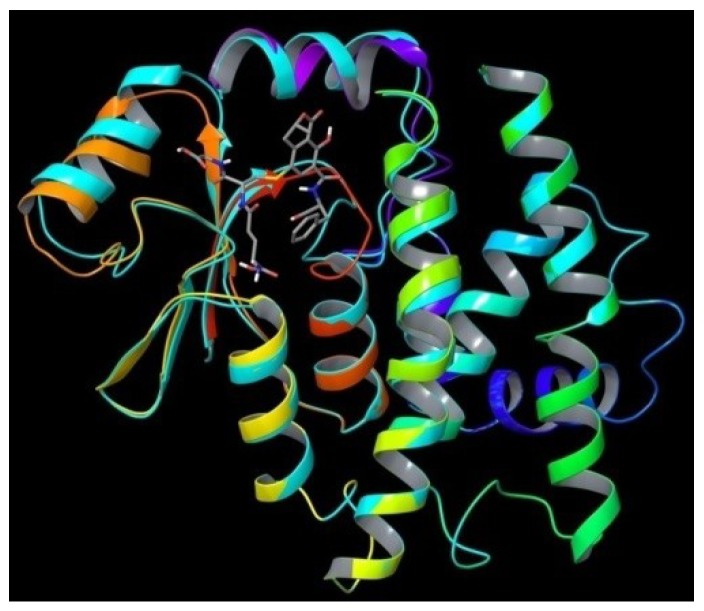
Superposition of glutathione transferase A1 (GSTA1) monomer with or without OTB-SG conjugate (A1). Free/ligand protein structure is colored in cyan.

As the result of *in silico* modeling, we obtained four structures, two for OTB-SG (A1 and A2) and two for OTHQ-SG (B1 and B2), bound in a GSTA1 monomer. Structures of ligands, which were placed in the binding site, are given on Figure 1. The structures of protein monomer was superimposed with structures of bound conjugates. GSTA1 protein is able to bind all four conjugates without significant changes in its structure (Figure 2 and Figures S1–S3, free/ligand protein structure is colored in cyan). The main changes are in the position of the two upper helices that are mobile (*C*-terminal helix 8, colored in magenta). The total potential energies of all the structures after the final minimization are given in Table S1.

The results of this study, performed in 207 BEN patients and 138 non-BEN individuals from an endemic region, have shown that carriers of at least one GSTA1*B lower expression allele independently and in combination with the *GSTT1* *active* allele are at higher risk of BEN development. The other two common *GST* polymorphisms tested, *GSTM1* and *GSTP1*, were not significantly associated with BEN. *In silico* analysis showed that GSTA1 might be involved in catalyzing reactions of OTA metabolites with GSH.

Our hypothesis that *GST* polymorphisms might result in differential susceptibility to BEN is based on the fact that GSTs catalyze several types of reactions that may be relevant for metabolism of OTA, one of the putative causes of BEN. It should be noted that until now only two studies have investigated the association between common *GST* gene variants and BEN risk. Andonova *et al*. [20] determined *GSTM1*, *GSTT1*, and *GSTP1* polymorphisms in 54 BEN patients and 104 endemic controls and found *GSTM1* *active* to be more prevalent among patients than controls. In another Bulgarian study, which was performed in 95 BEN patients and 112 non-endemic controls, the frequency of the *GSTM1* *active* genotype was also higher in BEN patients than in controls, but did not reach statistical significance [21]. The results of our study, obtained in a much larger cohort of BEN patients, failed to show any difference in the distribution of *GSTM1* gene variants between patients and controls, suggesting that this member of cytosolic GSTs is probably not involved in the metabolism of compounds responsible for BEN occurrence. It should be emphasized that regarding active *GST* gene variants tested in this study, only the *GSTT1* *active* genotype was more frequent among BEN patients than controls. The risk of BEN was 1.5 times higher in persons with *GSTT1* *active* genotype in comparison to *GSTT1 null* individuals, but this OR did not reach statistical difference. Our results are in accordance with data of Lebrun *et al.* [22], who pointed out the role of *GSTT1* polymorphism in the extent of DNA damage induced by OTA. Namely, the *GSTT1* *active* genotype was more frequently observed in the group of human donors of urothelial cells with DNA damage compared to a subgroup without DNA damage, both treated with OTA. In addition, the *GSTT1* *active* genotype is frequently associated with exposure to occupational hazards, such as trichloroethylene TCE [23], smoking habits [24], and pesticides [25]. Although the fact that the *GSTT1* *active* genotype confers the risk of BEN, the GSTT1-1 mediated conjugation of OTA with GSH into OTB-SG seems to be hardly possible. Namely, due to the hindered GSTT1 active site [26], large molecules like OTA cannot be accommodated. Therefore, the role of *GSTT1* polymorphism in ochratoxin nephrotoxicity still has to be clarified.

The most prominent result of our study is that the presence of the *GSTA1* gene variant, which results in lower transcriptional activation, is associated with a higher risk of BEN. It should be noted that the presence of two mutant GSTA1*B alleles results in a four times lower expression of this enzyme in the liver. GSTA1-1 is one of the most promiscuous GST enzymes with wide substrate specificity. It catalyses basically two types of reactions including conjugation with GSH and peroxidase reactions, in which GSH acts as a co-substrate to reduce organic hydro-peroxides. GSTA1-1 acts as a key liver cytosolic enzyme regarding GSH-dependent detoxification and antioxidant protection, especially in *GSTM1* *null* individuals who do not express GSTM1-1 protein. Similarly, GSTA1-1 plays a fundamental role in renal tissue protection, from endproducts of both endogenous and exogenous compounds metabolisms, specifically in proximal tubules, where it is abundantly expressed [27]. Regarding OTA nephrotoxicity, it seems that both GSH-conjugation and antioxidant defense catalyzed by GSTA1-1 might play distinct roles in the kidney. The reactions mediated by GSTA1-1 are supposed to result in lower OTA toxicity. Therefore, it seems reasonable to assume that GSTA1-1 is directly involved in the conjugation of OTA or its metabolite. Having in mind that higher expression of GSTA1-1 in individuals with the GSTA1*A/*A genotype is associated with lower BEN risk. Therefore, we assumed that GSTA1-1 would most probably catalyze OTB-SG, as well as the formation of OTHQ-SG (Chart 2). Indeed, based on our *in silico* analysis, both covalent adducts could be obtained as GSTA1-1 products.

The protective role of GSTA1-1 in terms of OTA toxicity and BEN risk is much easier to explain in the context of powerful antioxidant activity of GSTA1-1. Recent and most recent studies clearly establish a link between oxidative stress and OTA metabolism [28,29,30]. Thus, it seems that OTA influences both increased free radical production and decreased the gene expression of enzymes involved in glutathione synthesis and utilization [31,32]. Among OTA metabolites, OTHQ exerts strong pro-oxidant activity. Like other hydroquinones, OTHQ undergoes oxidation to generate superoxide and the quinone electrophile OTQ [10]. In the human renal proximal tubular epithelial cell line (HK-2), it was found that OTA treatment led to the release of reactive oxygen species and the increase of 8-hydroxydeoxyguanosine, an important biomarker of oxidative DNA damage [33]. Therefore, it seems reasonable to assume that the lack of GSTA1-1 antioxidant activity in conditions of chronic OTA exposure would lead to increased oxidative stress in kidney tissue. In this line, our data are in favor of the recent hypothesis of Cavin *et al.* [34], who supposed that failure of antioxidant protection mediated by GSTs promoted OTA-induced toxicity and oxidative stress. Moreover, the same group showed that OTA itself, at concentrations that are manifold higher than those detected in human and animal plasma, down-regulates overall GST expression and activity by inhibition of redox sensitive erythroid 2-like 2 (Nrf2) transcription factor in the kidney tubules cell line LLC-PK1 [35]. Nrf2 inhibition results in down-regulated expression of key enzymes of glutathione synthesis and metabolism, such as glutamine-cysteine ligase (GCL) and GSTP1-1 [31,36]. Although Nrf2 is not involved in the regulation of *GSTA1* expression [37], a decreased GSTA1-1 protein level of GSTA1*B homozygotes might deepen the prooxidant-antioxidant disbalance in BEN. In the future, it would be important to address whether these individuals exhibit higher oxidative stress and if treatment with antioxidants (e.g., intact glutathione) could slowdown BEN progression. It should be emphasized that in addition to its catalytic role, GSTA1-1 has also been shown to exhibit several non-catalytic functions, among which its anti-apoptotic activity has recently started to emerge, especially in context of anti-tumor drug resistance. In the view of the pathological changes that occur in the course of BEN progression, such as cell shrinking, tubular atrophy, and diffuse fibrosis of cortical interstitium, which further lead to reduction in the size of the kidney and increased apoptosis [38,39], lowered GSTA1-1 expression would be expected to result in more apoptosis. GSTA1-1 acts as modulator of mitogen-activated protein kinase signal transduction pathways via a mechanism involving protein-protein interactions. GSTA1-1 forms complexes with c-Jun *N*-terminal kinase (JNK) and modifies JNK activation during cellular stress, but the factors that influence complex association and dissociation are unknown. Very recently, it has been suggested that the mechanism of menadione-induced JNK activation involves the production of reactive oxygen species, likely superoxide anion, and intracellular GSH levels play an important role in preventing GSTA1-1-JNK complex dissociation, subsequent JNK activation, and induction of cytotoxicity [40]. Having in mind the presence of pro-oxidant OTA derivatives, as well as increased oxidative stress in BEN, such mechanisms might also take place in the case of BEN progression. Results of Romero *et al*. [41] have indicated that GSTA1-1 suppresses activation of apoptotic JNK signaling by a pro-inflammatory cytokine and oxidative stress and suggests a protective role for GSTA1-1 in JNK-associated apoptosis in MEF3T3 cells. Therefore, the assumption that GSTA1-1 might also play some functional non-catalytic role in BEN development should be addressed in the future.

## 3. Experimental Section

### 3.1. Study Participants

This case-control study comprised 207 patients with Balkan endemic nephropathy and 138 healthy controls, residents of endemic settlements with a negative family history for BEN. The BEN group consisted of patients who fulfilled criteria for BEN according to Stefanovic *et al.* [42], after exclusion of other kidney diseases. All patients with BEN were recruited in two hemodialysis centers from Bosnia and Herzegovina—Republic of Srpska (Bijeljina and Šamac) and one center from the Republic of Serbia (Lazarevac). Gender- and age-matched (±2 years) healthy subjects from non-BEN families were registered during the screening studies carried out in endemic villages in the same regions from January 2012 to December 2012. Subjects invited to the control group were included in this study only if no pathological finding was detected by objective examination and routine laboratory analysis. Demographic and clinical characteristics were obtained from medical records. All the participants provided written informed consent. This study protocol was approved by the Institutional Review Board (permission number 29/VI-13), and the research was carried out in compliance with the Helsinki Declaration (as revised in 2000).

### 3.2. GST Genotyping

Genomic DNA was isolated from whole blood using the QIAmp^®^ DNA Blood Mini kit (Qiagen, Inc., Chatsworth, CA, USA) according to the manufacturer’s protocol.

The analysis of *GSTA1 C-69T* SNP was performed using the polymerase chain reaction-restriction fragment length polymorphism (PCR-RFLP) method, as described previously by Ping *et al*. [43]. A 400 bp fragment of *GSTA1* gene was amplified with primers: *GSTA1* forward primer 5'-GCATCAGCTTGCCCTTCA-3' and *GSTA1* reverse primer 5'-AAACGCTGTCACCGTCCTG-3'. The presence of the Eam1104I restriction site yielded two fragments of 308 and 92 bp (GSTA1*B), whereas, the absence of restriction site was determined by the presence of a 400 bp fragment (GSTA1*A).

*GSTM1* and *GSTT1* deletion polymorphisms were determined by multiplex PCR method, as described by Abdel-Rahman *et al*. [44], with some modifications. The following primers used were: *GSTT1* forward primer 5'-TTCCTTACTGGTCCTCACATCTC-3'; *GSTT1* reverse primer 5'-TCACCGGATCATGGCCAGCA-3'; *GSTM1* forward primer 5'-GAACTCCCTGAAAAGCTAAAGC-3'; *GSTM1* reverse primers: 5'-GTTGGGCTCAAATATACGGTGG-3', and instead of the *CYP1A1* gene as an internal positive control, *β-globin* gene primers were used: 5'-ACACAACTGTGTTCACTAGC-3' and 5'-CAACTTCATCCACGTTCACC-3' [45]. Using these primers, the assay does not distinguish between heterozygous or homozygous wild type genotypes; therefore, the presence of 215 and 480 bp bands were indicative for the *GSTM1* and *GSTT1active* genotype, respectively. Amplification of the *β-globin* gene was used as the internal positive control and produced a 110 bp fragment.

*GSTP1 A1578G* (*Ile105Val*) polymorphism was determined by the previously described PCR-RFLP method [46]. *GSTP1* forward primer 5'-ACCCCAGGGCTCTATGGGAA-3' and *GSTP1* reverse primer 5'-TGAGGGCACAAGAAGCCCCT-3' were used to amplify a 176 bp fragment of *GSTP1* gene. After digestion, fragments were separated and three different patterns were obtained: the wild genotype *Ile*/*Ile* had only a 176 bp band, the homozygous mutant genotype *Val*/*Val* is characterized by 91 and 85 bp products, while the heterozygous genotype *Ile*/*Val* yielded three bands (176, 91 and 85 bp).

Because the assays are extremely robust, quality control procedures were implemented as random repeats, water blanks for checking DNA contamination, and orientation markers. Results of genotyping for the study were read from the gel by two independent investigators, and at least 20% of the samples were randomly repeated.

### 3.3. Statistical Analysis

In the descriptive statistics, we report continuous variables by mean ± standard deviation (SD). We used one-way ANOVA for comparing the means of continuous variables. The chi-square (χ2) test was used to compare the gender distribution and to test the deviation of genotype distribution from the Hardy-Weinberg equilibrium (HWE). Also, the statistical significance of the differences between the frequency genotypes of groups was determined using the chi-square test. Two-sided *p* < 0.05 was considered to be statistically significant.

Binomial logistic regression was used to estimate the odds ratio (OR) and 95% confidence intervals (CI) with two-sided p-values for the association between BEN and the studied genotypes, adjusted for age and gender.

The SPSS 17.0 statistical software package (SPSS Inc., Chicago, IL, USA) was used to assess the significance of the studied variables.

### 3.4. Molecular Modeling (in Silico)

The crystal structure of human glutathione transferase A1-1 bound with chlorambucil was downloaded from the protein data bank (http://www.rcsb.org/, PDB ID 4HJ2) [47]. The protein structure was prepared by deleting water molecules and reducing enzymes into a monomer structure. OTA metabolites were covalently bonded to bound GSH, and OTHQ-SG and OTB-SG were produced. Conjugates were placed in two starting positions: one with the peptide part of the subunit facing the inner part of the protein and another with the peptide part of subunit facing the solvent accessible space of the enzyme. All structures were subjected to a minimization procedure in Impact (version 5.8, Schrödinger, L.L.C., New York, NY, USA, 2005), using OPLS2005 force field and the Surface Generalized Born (SBG) solvation model. During minimization, the following constraints were applied: all amino acids occupying a space of more than 18 Å from the ligand were kept frozen and the glutathione part of the conjugate was subjected to a constraint of 25.0 kcal/mol force. Truncated Newton algorithm with 100 maximum cycles was applied. The resulting structures were furthermore subjected to an Impact Dynamics procedure, using the same force field and solvation model and constraints, with other parameters set as default values. After that, the final structures were again minimized under the same conditions, but this time with 2000 maximum cycles and with the energy and gradient convergence criteria set to 1 × 10^−7^ kcal/mol and 0.01 kcal/(mol Å), respectively.

## 4. Conclusions

We found that *GSTA1* polymorphism is associated with an increased risk of BEN.

## Supplementary Files

Supplementary File 1
