# Supplementary material for: Is Increased Susceptibility to Balkan Endemic Nephropathy in Carriers of Common GSTA1 (*A/*B) Polymorphism Linked with the Catalytic Role of GSTA1 in Ochratoxin A Biotransformation? Serbian Case Control Study and In Silico Analysis
Source: Toxins (Basel). 2014 Aug 8;6(8):2348–62. doi: 10.3390/toxins6082348 (PMC4147586; doi:10.3390/toxins6082348)
Supplement: Supplementary File 1 [file toxins-06-02348-s001.pdf]

Supplementary Information

Figure S1. Superposition of glutathione transferase A1 (GSTA1) monomer with or without OTB-SG conjugate (A2). Free/ligand protein structure is colored in cyan.

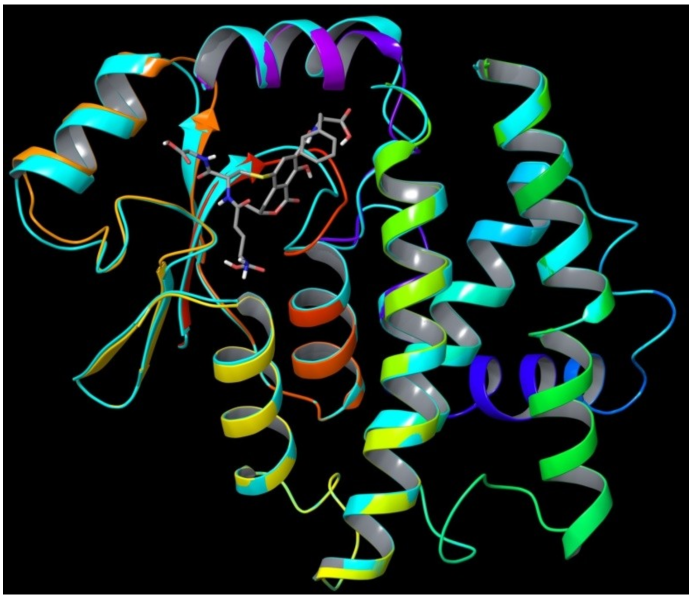

Figure S2. Superposition of glutathione transferase A1 (GSTA1) monomer with or without OTB-SG conjugate (B1). Free/ligand protein structure is colored in cyan.

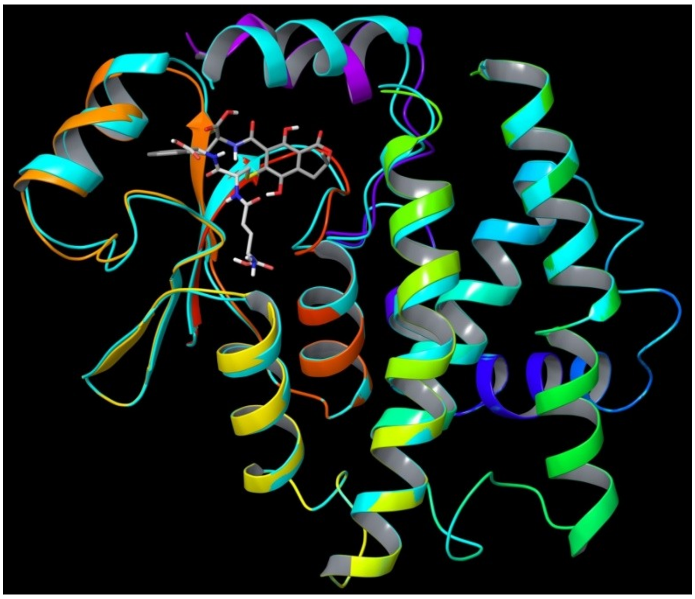

Figure S3. Superposition of glutathione transferase A1 (GSTA1) monomer with or without OTB-SG conjugate (B2). Free/ligand protein structure is colored in cyan.

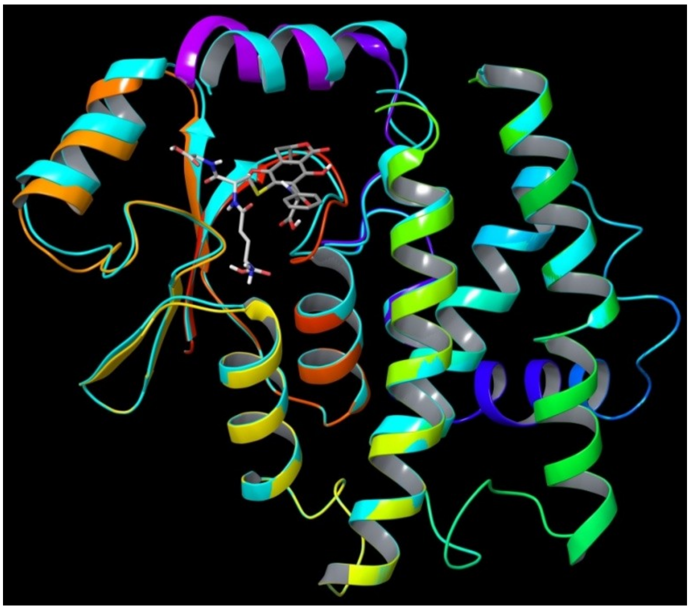

Table S1. Calculated total potential energy (kcal/mol) of optimized conjugates A1, A2, B1 and B2.

Structure	Total potential energy (kcal/mol)
A1	−7879.17
A2	−8268.93
B1	−7760.40
B2	−8019.00
